# Highly Significant Association between Two Common Single Nucleotide Polymorphisms in *CORIN* Gene and Preeclampsia in Caucasian Women

**DOI:** 10.1371/journal.pone.0113176

**Published:** 2014-12-04

**Authors:** Alain Stepanian, Alexandre Alcaïs, Dominique de Prost, Vassilis Tsatsaris, Michel Dreyfus, Jean-Marc Treluyer, Laurent Mandelbrot, on behalf of the ECLAXIR Study Group

**Affiliations:** 1 Service d’Hématologie Biologique, Hôpital Lariboisière, Paris, France; 2 Laboratory of Human Genetics of Infectious Diseases, Necker Branch, Institut National de la Santé et de la Recherche Médicale, U1163, Imagine Institute, Paris, France; 3 University Paris Descartes, Sorbonne Paris Cité, Paris, France; 4 St Giles Laboratory of Human Genetics of Infectious Diseases, Rockefeller Branch, The Rockefeller University, New York, New York, United States of America; 5 Service d’Hématologie biologique et Transfusion, Hôpital Louis Mourier (AP-HP), Colombes, France; 6 Unité 770, INSERM, Le Kremlin-Bicêtre, France; 7 Service de Gynécologie-Obstétrique, Hôpital Port-Royal (AP-HP), Paris, France; 8 Unité 767, INSERM, Université Paris Descartes, Paris, France; 9 Département Hospitalo-Universitaire Risque et Grossesse, Paris, France; 10 Fondation PremUP, Paris, France; 11 CHU Clémenceau, Unité de Gynécologie-Obstétrique et Médecine de la Reproduction, Caen, France; 12 INSERM CIC P0901, Paris, France; 13 Université Paris Diderot, Sorbonne Paris Cité, Paris, France; 14 AP-HP, Hôpital Louis Mourier, service de Gynécologie-Obstétrique, Colombes, France; David Geffen School of Medicine at UCLA, United States of America

## Abstract

Preeclampsia is a frequent medical complication during pregnancy. Corin, a serine protease which activates pro-atrial natriuretic peptide, has recently been shown to be involved in the pathophysiology of preeclampsia. The aim of this study was to search for *CORIN* gene variations and their association to preeclampsia in Caucasian and African women. Our study population was composed of 571 pregnant women (295 with preeclampsia and 276 normotensive controls) matched for maternal and gestational age, and ethnic origin. The 22 exons of the *CORIN* gene were sequenced in a discovery sample (n = 260), where 31 single nucleotide polymorphisms were identified. In a replication sample (n = 311), 4 single nucleotide polymorphisms were tested. Two minor alleles (C for rs2271036 and G for rs2271037) were significantly associated to preeclampsia. Adjusted odds ratios [95% confidence interval] were 2.5 [1.2–3.8] (*p* = 0.007) and 2.3 [1.5–3.5] (*p* = 1.3×10^−4^), respectively. These associations were ethnic-specific, as only found in the Caucasian of subjects (odds ratio = 3.5 [1.8–6.6], *p* = 1.1×10^−4^; odds ratio = 3.1 [1.7–5.8], *p* = 2.1×10^−4^, for each single nucleotide polymorphism, respectively). The two single nucleotide polymorphisms are in almost perfect linkage disequilibrium (r^2^ = 0.93). No specific association was found with severe preeclampsia, early-onset preeclampsia nor fetal growth retardation. In conclusion, this is the first report of a highly significant association between these two single nucleotide polymorphisms in *CORIN* gene and preeclampsia. Our findings further support the probability of a critical role of corin in preeclamspia pathophysiology at the uteroplacental interface.

## Introduction

Preeclampsia (PE) is a frequent medical complication during pregnancy, occurring in 2% to 7% of pregnancies worldwide [1], [2]. It is an important cause of perinatal and maternal mortality and morbidity, in particular preterm birth. Diagnosis is based on hypertension occurring after 20 weeks of gestation, associated with significant proteinuria [3]. Despite extensive research, the initial pathological processes remain elusive. The earliest known abnormality is defective trophoblast invasion of the placenta, which disrupts uterine artery morphology and leads to placental hypoxia and ischemia. This is accompanied by the secretion by the syncytiotrophoblast of several soluble mediators into maternal circulation, some of which promote inflammation and result in maternal endothelial cell injury [4]–[6]. PE is sub-classified in early- or late-onset PE based on the time of diagnosis (before or after 34 weeks). There is evidence suggesting that these two sub-groups of PE might result from different mechanisms, with placental pathology being predominant in early-onset PE and maternal inflammatory state being predominant in late-onset PE [4], [7].

Corin is a transmembrane type II serine protease expressed primarily in cardiomyocytes, were it cleaves pro-atrial natriuretic peptide (ANP) into active ANP, a cardiac hormone that regulates blood pressure [8], [9]. Corin is supposed to be involved in placental development and in the pathophysiology of preeclampsia. Interestingly, corin expression was detected in decidual cells of pregnant mice, as well as in human endometrial cells [10]. Pregnant *CORIN* knockout mice develop significant increase of systolic blood pressure associated with late-gestational proteinuria, mimicking human PE phenotype [11]. These corin-deficient mice also have smaller uterine spiral arteries than wild-type mice, pointing to impaired trophoblast invasion and spiral artery remodeling [12]. In preeclamptic patients, uterine levels of corin mRNA and protein were significantly lower than those in normal pregnant women [12].

Human *CORIN* gene is located on chromosome 4p12-p13, has 22 exons and spans 244 kb [13]. Two corin variants (Q568P and T555I) in perfect linkage disequilibrium (LD), i.e. describing in reality a single minor corin gene allele defined by the presence of both rare alleles I555 and P568 on the same parental chromosome, and more common in African-Americans than in Caucasians are associated with hypertension [14]. In preeclamptic patients from China, two other mutations were detected: Lys317Glu in the LDLR2 domain and Ser472Gly in the Frizzled 2 domain, which are important for ANP processing [12], [15]. In functional experiments, both corin mutants exhibited markedly reduced pro-ANP processing activities [12].

To date, there are no data on *CORIN* gene variations in large series of human patients with preeclampsia. The aim of our study was to search for *CORIN* gene variations and their frequency in Caucasian and Sub-Saharan African pregnant women with and without preeclampsia.

## Materials and Methods

### Patients

In this study, we used the samples of the “Search for an Association between CX3CR1 and V249I Polymorphisms, Preeclampsia and Endothelial Injury” (ECLAXIR) study, a multicenter case-control study. The objectives and design of the ECLAXIR study are detailed in a previous work where 2 polymorphisms of CXR3R1 were analyzed [16]. The study was approved by the Ethics Committee (Comité de Protection des Personnes dans la Recherche Biomédicale, CCPPRB) of the Bichat-Claude Bernard hospital (Paris). The patients were recruited from 6 French university hospitals between May 2003 and October 2007, as previously reported [16]. The cases were pregnant women with PE at time of diagnosis, originated from two ethnic groups, Caucasians from Europe (mainly from France) or from the Maghreb (Morocco, Algeria and Tunisia) and Africans (Sub-Saharan Africa). The controls were pregnant women without PE. Each case was matched with a control; the matching criteria were ethnic origin as defined by the region of birth of all four grandparents (Europe, Maghreb and Sub-Saharan Africa), maternal age±3 years and gestational age±2 weeks. Controls who developed PE after inclusion were excluded.

PE was defined as blood pressure ≥140/90 mm Hg occurring after 20 weeks of gestation with previously normal blood pressure, associated with proteinuria ≥0.3 g in a 24-hour urine specimen [3]. Severe PE was defined according to the American College of Obstetricians and Gynecologists criteria [3] as the presence of at least one of the following: blood pressure ≥160/110 mm Hg in 2 measurements 4 hours apart while the patient was on a bed rest; proteinuria ≥5 g in a 24-hour urine specimen; oliguria of less than 25 mL per hour; cerebral or visual disturbances; pulmonary edema or cyanosis; epigastric pain; impaired liver function defined as serum aspartate aminotransferase concentrations ≥70 IU/L; thrombocytopenia defined as platelet count lower than 100 Giga/L; fetal growth restriction (FGR). We selected a cut-off for FGR below the third percentile, equivalent to a *z*-score lower than −1.88, calculated using tables from a Paris area-based population where birthweight was adjusted for gestational age and sex [17]. Early PE was defined as a gestational age at diagnosis <34 weeks. Obesity was defined as pre-pregnancy body mass index (BMI) >30 kg/m^2^.

Cases and controls were recruited as pairs, but as a few subjects were excluded (e.g. controls who developed PE after inclusion, missing samples…), our final sample was composed of pairs and supplemental unmatched cases. These latter were rematched with one pair using the same criteria as the ones described above. Finally, our study population was divided into 2 samples of nearly same size. In the discovery sample (sample 1), composed of randomly chosen subjects, we searched for genetic variations by sequencing the whole 22 exons of *CORIN* (see below), and tested their association with PE. In the replication sample (sample 2), composed of the remaining patients of the ECLAXIR study, we genotyped the most promising Single Nulceotide Polymorphiss (SNPs) identified in the discovery sample, to replicate their association with PE, and strengthen the evidence for a true effect.

### Blood sampling and DNA extraction

Written informed consent was obtained from each woman before enrolment and blood sampling. Venous blood was collected in 15% K_3_EDTA solution, at the time of enrolment (*i.e.* at time of diagnosis for patients, before any treatment). DNA extraction was performed with QIAmp DNA Blood Midi Kit (Qiagen^©^, Courtaboeuf, France), according to the manufacturer’s instructions. The samples were also stored at −80°C until testing.

### Sequencing and Genotyping

As a first step, amplification of the 22 exons of the *CORIN* gene was first performed in a discovery sample (sample 1) composed of randomly chosen patients within the ECLAXIR study. This was done by means of the VariantSEQr System. The VariantSEQr System protocol consists of 5 steps: (1) PCR amplification of human genomic DNA, (2) purification of PCR product, (3) cycle sequencing, (4) electrophoresis on a sequencer, and (5) data analysis. PCR primers used for the amplification of the amplicon contain a specific sequence of the exon and either universal primer M13 forward (M13F) or M13 reverse (M13R) sequences for subsequent sequencing reaction; all sequences are freely available on www.pubmed.org [Probe section - *Homo sapiens* RSA (ReSequencing Amplicon) probe]. PCR conditions were defined as: 1 µL of DNA (30 ng/µL), 0.45 µL of each primers (10 µM), 0.15 µL of dNTP (25 mM each), 0.6 µL of MgCl2 (50 mM), 1.5 µL of (50 mM KCl, 20 mM Tris HCl (pH: 8.4)), 3 µL of 40% Glycerol in water, 0.15 µL of Taq, water 8.5 µL. All PCR were performed as followed: (i) denaturation: (96°C - 5 min) on initial cycle and (94°C - 30 sec) on rest; (ii) annealing: (60°C - 45 sec); (iii) extension: (72°C - 47 sec - 43 cycles) and 13 min on last cycle. PCR were checked on gel and purified by Sepadex G50. Sequencing was performed by a single universal primer (M13R or M13F). Specifically, a total of 8 kb (exons and intronic regions flanking the exons) were amplified from each of the genomic DNA samples, and sequenced using a capillary-based ABI 3730xl DNA Analyzer (BigDye Terminator cycle sequencing kits v3.1; Life Technologies, Grand Island, New York, USA). Sequence traces were assembled and aligned for variant calling using Lasergene SeqMan v8.1 (DNASTAR, Madison, Wisconsin, USA). The variants were oriented according to Human Genome 19.

In a second step, the two most promising SNPs identified in the discovery sample were subsequently genotyped as described above in the replication sample (sample 2), composed of the remaining patients of the ECLAXIR study.

Phylogenetic analysis, i.e. comparison of Corin protein sequence with other species, was performed using the Ortheus algorithm as implemented in the ensembl platform (www.ensembl.org).

### Statistics

Descriptive demographic and clinical data were expressed in percentages or means ± standard deviations (SD). Conditional logistic regression analysis performed with SAS was used to compare qualitative and quantitative variables between cases and controls in both sample 1 and sample 2, and in the combined sample. We estimated population allelic frequencies, pairwise LD (expressed as r^2^) between SNPs and tested for Hardy Weinberg equilibrium using the algorithm implemented in Haploview 4.2 [18], [19]. SNPs with minor allelic frequency (MAF) <1% were not further analyzed. Conditional logistic regression analysis was performed with the LOGISTIC procedure of the SAS software v9.2 (SAS institute, Cary, NC) and results were expressed as odds ratios (ORs) with 95% confidence intervals (CI)s for the most significant genetic model (additive, dominant or recessive), in both samples and in total combined sample. All analyses were adjusted on BMI and nulliparity since these covariables were significantly associated with the onset of PE in our sample (see results). Multiple testing issue was accounted for by means of the stringent Bonferroni correction and a test was considered significant if the p-value was lower than 0.05/14 = 0.004 (0.05 divided by the number of SNPs kept for further analysis after quality check, i.e. 14).

## Results

### Identification of the polymorphisms and characteristics of the study population

**Figure 1:**
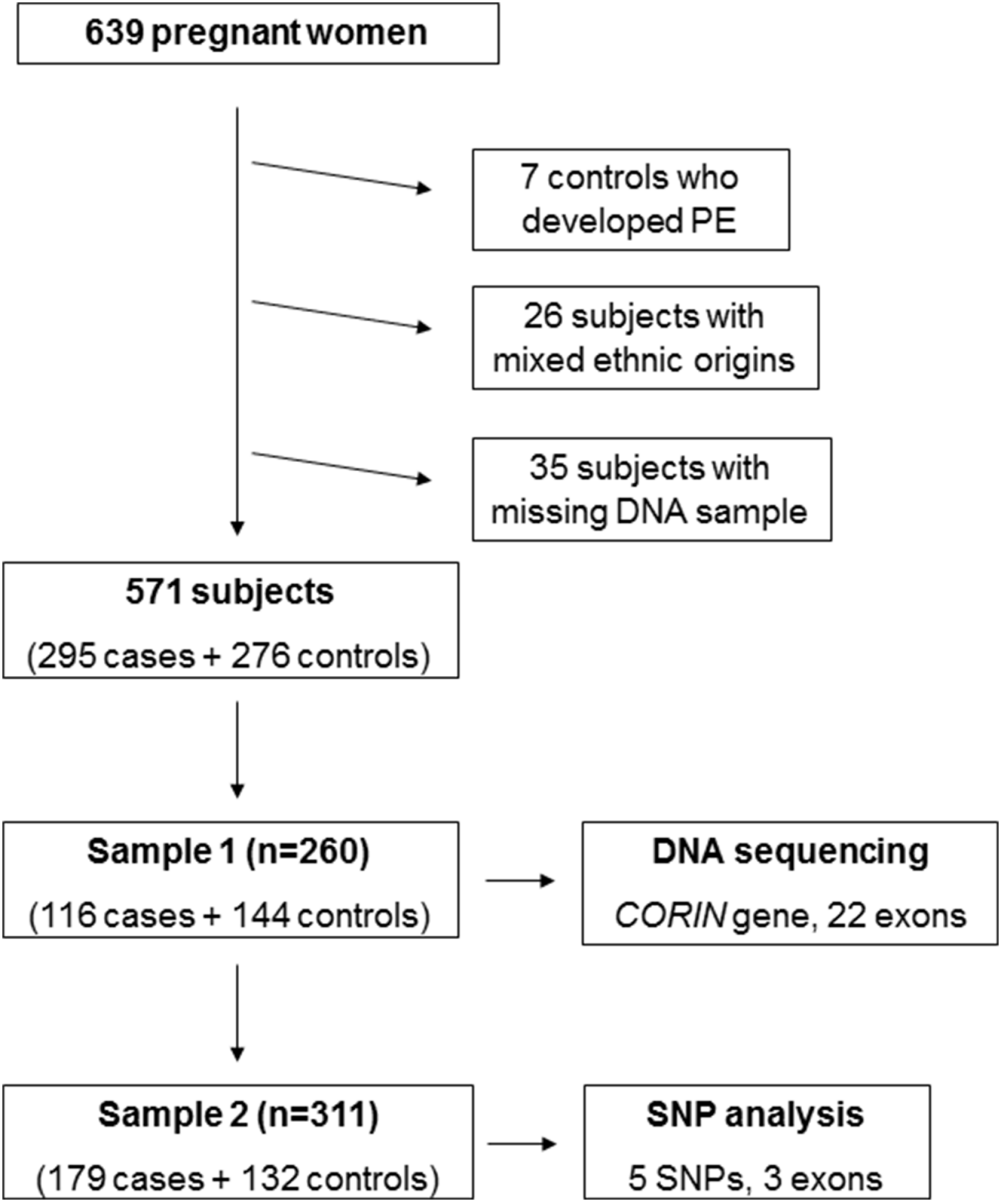
Flow chart for the enrollment of the study population and the SNP analysis.

We included 639 patients. Seven controls who developed PE after enrollment, 26 patients with unmatchable mixed ethnic origins and 35 patients with insufficient amounts of DNA for genotyping were excluded from the analysis. In the current study, we analyzed 571 matched subjects (295 cases and 276 controls) (Figure 1).

Table 1 shows general demographic, obstetrical and medical characteristics of both samples 1 and 2 and total population. No systematic difference was found significant between these two samples. Obesity and nulliparity were significantly more frequent in preeclamptic patients than in controls (20.0% *vs.* 8.0% [*p*<0.0001] and 41.3% *vs.* 31.5% [*p* = 0.006], respectively). As expected, the proportion of neonates small for gestational age (SGA) was higher in patients with PE as compared to controls. There were no significant differences between cases and controls for history of thrombosis, pregestational diabetes, primipaternity, connective tissue disease or antiphospholipid antibody.

**Table 1:** Demographic, obstetrical and medical characteristics of the study population.

	Sample 1		Sample 2		Combined sample		*p* value*	*p* value†
	(n = 260)		(n = 311)		(n = 571)			
	Cases	Controls	Cases	Controls	Cases	Controls		
	(n = 116)	(n = 144)	(n = 179)	(n = 132)	(n = 295)	(n = 276)		
**Age (years) -** mean ± SD	31.6±6.1	30.7±5.6	30.2±6.1	31.2±5.8	30.8±6.1	30.9±5.7	-	-
**Caucasian from Europe** % (n)	32.8 (38)	56.9 (82)	63.7 (114)	49.2 (65)	51.5 (152)	53.3 (147)	-	-
**Caucasian from Maghreb** % (n)	18.1 (21)	13.9 (20)	15.6 (28)	21.2 (28)	16.6 (49)	17.4 (48)	-	-
**Sub-Saharan African** % (n)	49.1 (57)	29.2 (42)	20.7 (37)	29.5 (39)	31.9 (94)	29.3 (81)	-	-
**Gestational age (weeks)** **-** mean ± SD	33.3±4.2	33.3±4.1	33.6±4.2	33.1±4.2	33.5±4.2	33.2±4.1	-	-
**BMI before pregnancy** **>30** **kg/m^2^** % (n)	19.0 (22)	8.3 (12)	20.7 (37)	7.6 (10)	20.0 (59)	8.0 (22)	<10^−4^	<10^−4^
**Nulliparity** % (n)	35.3 (41)	30.6 (44)	45.2 (81)	32.6 (43)	41.3 (122)	31.5 (87)	0.007	0.006
**Primipaternity** % (n)	10.6(10/94)	9.2(12/131)	16.4(25/152)	12.1(14/116)	14.2(35/247)	10.5(26/247)	0.2	0.5
**Multiple pregnancy** % (n)	8.6 (10)	3.5 (5)	6.1 (11)	0	7.1 (21)	1.8 (5)	0.006	0.07
**Small for gestational age** % (n)	14.7 (17)	1.4 (2)	12.3 (22)	3.8 (5)	13.2 (39)	2.5 (7)	<10^−4^	<10^−4^
**Personal history of venous** **thrombosis** % (n)	2.6 (3)	0	1.1 (2)	0.8 (1)	1.7 (5)	0.4 (1)	0.1	NS
**Familial history of venous** **thrombosis** % (n)	2.6 (3)	4.9 (7)	10.6 (19)	3.8 (5)	7.5 (22)	4.3 (12)	0.2	NS
**Previous preg. loss before** **22 GW** % (n)	26.7 (31)	19.4 (28)	18.4 (33)	24.2 (32)	21.7 (64)	21.7 (60)	0.9	NS
**Previous foetal death after** **22 GW** % (n)	2.6 (3)	2.8 (4)	2.8 (5)	1.5 (2)	2.7 (8)	2.2 (6)	0.3	NS
**Pregestational diabetes** % (n)	2.6 (3)	0	2.2 (4)	0.8 (1)	2.4 (7)	0.4 (1)	0.1	NS
**Connective tissue disease** **or APLS** % (n)	3.4 (4)	0	2.2 (4)	2.3 (3)	2.7 (8)	1.1 (3)	0.3	NS

Variation in number is due to missing data.

*p value from univariate conditional logistic regression analysis.

† p value from multivariate conditional logistic regression analysis.

SD, standard deviation; BMI, body mass index; preg., pregnancy; GW, gestation weeks; APLS, antiphospholipid syndrome.

Fetal growth restriction, <3^rd^ percentile; NS, non significant.

The *CORIN* gene was first sequenced in the discovery sample 1 composed of 260 individuals (116 cases and 144 controls), where 31 polymorphisms wee identified. Twelve were disregarded because their minor allele frequency (MAF) was <1% in patients regardless of their ethnic origin (rs55932196, rs61759692, rs111721946, rs61760500, rs61760501, rs115431350, rs13359245, rs112649908, rs138148317, rs74701656, rs61764288 and rs113461950). Table 2 shows positions of the remaining 19 SNPs, with their MAF in Caucasian (n = 161) and Sub-Saharan-African (n = 99) sub-groups of subjects. Four of these SNPs were not analyzed because of a MAF ≤0.005 in the largest Caucasian group precluding a reasonably powered association test. None of the 19 SNPs displayed in Table 2 showed deviation from the Hardy-Weinberg equilibrium. Among the 14 SNPs studied, only two rs2271036 and rs2271037 were significantly associated to PE (p = 1.9×10^−5^ and p = 0.004, respectively) in the global sample. Among the remaining 12, all displayed a p-value above 0.2 but rs61759670 (*p* = 0.1).

**Table 2:** Quality control for the 19 *CORIN* single nucleotide polymorphisms (SNPs) found in the discovery sample (sample 1, n = 260).

SNP	Position	Allele		MAF		*p* (HWE)	
		Minor	Major	Caucasians*	Africans†	Caucasians*	Africans†
*rs73815721*	*47605421*	*C*	*G*	*0.0032*	*0.0260*	*1*	*1*
rs61759670	47605505	A	G	0.0350	0.0155	1	1
rs3215139	47625811	del	A	0.2546	0.2626	0.4256	1
rs1344122	47626034	T	C	0.2607	0.2677	0.4256	1
rs17462783	47643863	C	T	0.1074	0.1313	0.6214	0.1724
rs55821538	47645277	A	G	0.0215	0.0051	1	1
rs113780057	47647241	A	T	0.0263	0.0722	1	1
rs74503412	47655524	C	T	0.0184	0.1061	0.0721	0.1224
*rs77018190*	*47655556*	*C*	*T*	*0.0061*	*0.0404*	*1*	*1*
rs3805392	47655730	G	A	0.0729	0.0119	0.3833	1
*rs111253292*	*47663760*	*G*	*T*	*0*	*0.0758*	*1*	*1*
*rs75770792*	*47663799*	*G*	*T*	*0*	*0.0707*	*1*	*1*
rs11934749	47667064	T	C	0.1439	0.0114	0.3807	1
**rs2271036**	**47679928**	**C**	**T**	**0.2387**	**0.4714**	**0.1832**	**1**
**rs2271037**	**47680085**	**G**	**T**	**0.2594**	**0.68**	**0.0720**	**0.4445**
rs10517195	47682174	G	A	0.5024	0.2614	0.152	0.6164
rs149330314	47746257	del	TGT	0.0613	0.0773	1	0.1183
*rs111915728*	*47765389*	*A*	*G*	*0.0013*	*0.0417*	*1*	*0.1344*
rs2289433	47839929	C	T	0.2761	0.3889	0.634	0.7511

For each SNP, position on chromosome 4, minor allele frequencies (MAF) in both ethnic groups of subjects and results of testing for departure from Hardy Weinberg equilibrium (*p* (HWE)). Both SNPs of interest (rs2271036 and rs2271037) are in bold. SNPs with MAF below 1% in Caucasians are in italics.

del, deletion.

*Caucasian from Europe or Maghreb.

† Sub-Saharan African patients.

‡ Genotypic association with preeclampsia in a dominant model: conditional logistic regression analysis adjusted for nulliparity and obesity.

In a second batch of analysis, (sample 2, composed of 311 subjects: 179 cases and 132 controls) subjects were genotyped for the two SNPs most significantly associated with PE in sample 1 (i.e. rs2271036, rs2271037). Note that because of the genotyping technique, i.e. based on sequencing, genotypes were obtained for two additional SNPs in the vicinity of the same exon (rs11934749 and, rs10517195). Among them, only rs2271036 and rs2271037 were significantly associated to PE (p = 0.003 and 0.01, respectively see S2 lines in Table 3).

**Table 3:** Association of *CORIN* rs2271036 and rs2271037 single nucleotide polymorphisms (SNPs) with preeclampsia (PE).

SNP	Sample (n)	PE Cases	PE Cases	Controls	Controls	OR†	*p* †
Position		Genotypes% (n)*	MAF	Genotypes% (n)*	MAF	[95% IC]	
		**TT/TC/CC**		**TT/TC/CC**			
**rs2271036**	S1 (260)	35.3 (41)/50.9(59)/13.8 (16)	39.2‡	55.6 (80)/33.3(48)/11.1 (16)	27.8	2.7 [1.6–3.8]	1.9×10^−5^
**chr4.47679928**	S2 (311)	43.6 (78)/46.9(84)/9.5 (17)	33.0‡	57.6 (76)/35.6(47)/6.8 (9)	24.6	2.2 [1.4–5.4]	0.003
	C = S1+S2(571)	40.3 (119)/48.5(143)/11.2 (33)	35.4‡	56.5 (156)/34.4(95)/9.1 (25)	26.3	2.5 [1.2–3.8]	0.007
	**C-Eur** (299)	**50.0 (76)/44.7** **(68)/5.3 (8)**	**27.6** ‡	**68.7 (101)/25.9** **(38)/5.4 (8)**	**18.4**	**3.5 [1.8–6.6]**	**1.1×10^−4^**
	C-Mgh (97)	44.9 (22)/55.1(27)/0	27.5	56.3 (27)/37.5(18)/6.3 (3)	25.0	1.9 [0.7–5.0]	0.2
	C-Afr (175)	22.3 (21)/51.1(48)/26.6 (25)	52.1	34.6 (28)/48.1(39)/17.3 (14)	41.4	1.5 [0.7–3.4]	0.3
		**TT/TG/GG**		**TT/TG/GG**			
**rs2271037**	S1 (260)	25.0 (29)/50.9(59)/24.1 (28)	49.6‡	47.9 (69)/34.7(50)/17.4 (25)	34.7	2.8 [1.4–5.6]	0.004
**chr4.47680085**	S2 (311)	41.0 (73)/43.8(78)/15.2 (27)	37.1‡	50.0 (66)/27.3(36)/22.7 (30)	36.4	2.1 [1.2–3.8]	0.01
	C = S1+S2	34.7 (102)/46.6(137)/18.7 (55)	42.0‡	48.9 (135)/31.2(86)/19.9 (55)	35.5	2.3 [1.5–3.5]	1.3×10^−4^
	**C-Eur** (299)	**50.3 (76)/42.4** **(64)/7.3 (11)**	**28.5** ‡	**68.0 (100)/25.2** **(37)/6.8 (10)**	**19.4**	**3.1 [1.7–5.8]**	**2.1×10^−4^**
	C-Mgh (97)	34.7 (17)/63.3(31)/2.0 (1)	33.7	52.1 (25)/35.4(17)/12.5 (6)	30.2	2.3 [0.9–5.5]	0.07
	C-Afr (175)	9.6 (9)/44.7(42)/45.7 (43)	68.1	12.3 (10)/39.5(32)/48.1 (39)	67.9	0.8 [0.2–2.6]	0.7

Results are given in the discovery (S1), replication (S2) and combined (C) samples, in Caucasian subjects from Europe (Eur), Maghreb (Mgh) or Sub-Saharan-African subjects (Afr).

*Genotypes are expressed as percentage (number) of patients with TT/TC/CC genotype for rs2271036 and as TT/TG/GG genotype for rs2271037, respectively.

† ORs (95% IC, p values) calculated in a dominant model, adjusted for nulliparity and obesity, associated with the (CC+CT) versus the TT genotype (rs2271036) or with the (GG+GT) versus the TT genotype (rs2271037). MAF, minor allele frequency.

‡ Significantly different when compared to the control group (chi-square test adjusted for nulliparity and obesity).

**Figure 2:**
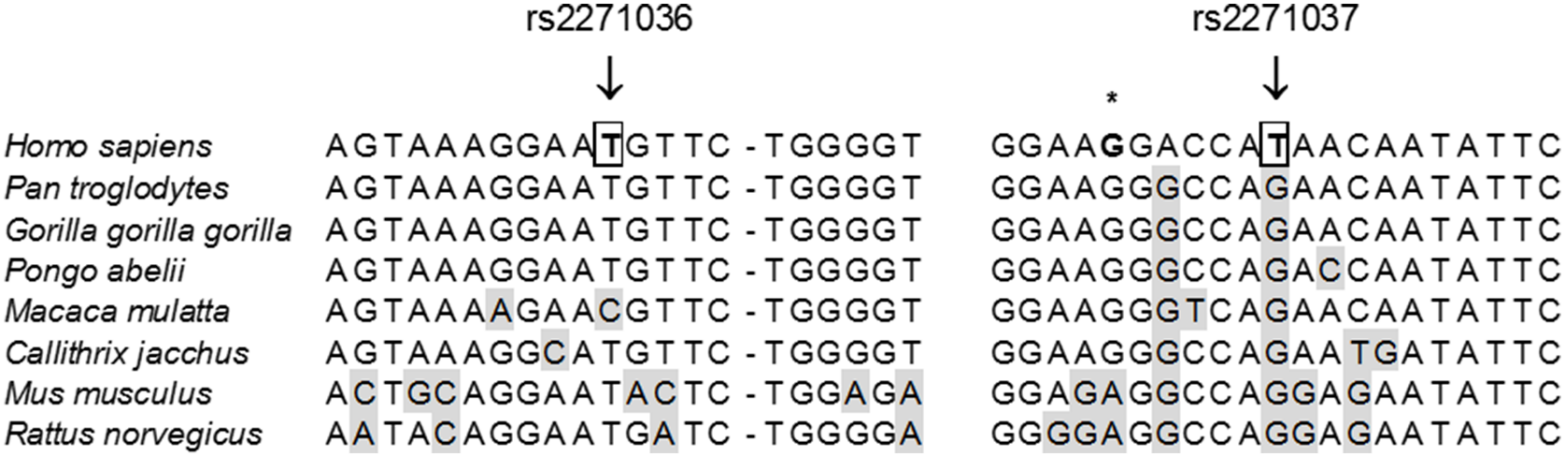
Comparison of human *CORIN* gene sequence with other species. Arrows on rs2271036 (left) and rs2271037 (right) locations at 4∶47679928 and 4∶47680085, respectively, in intronic regions flanking exon 9. The SNPs change the nucleotide indicated in bold and squared. Nucleotides are highlighted in grey when differing from human species. * above a splice region.

rs2271037 and rs2271036 SNPs are located immediately before and after exon 9 in *CORIN* at positions 4∶47680085 and 4∶47679928, respectively. As shown on Figure 2, the comparison of human corin nucleotide sequence with other species shows that both rs2271036 and rs2271037 are highly conserved within mammals and that rs2271037 is located closely to a splice region.

### Association between *CORIN* variants and preeclampsia

Table 3 shows the association between rs2271036 and rs2271037 SNPs and PE in sample 1, sample 2 and total combined study population. Our results showed significant differences in genotype frequencies between cases and controls in particular under a dominant model: the adjusted OR [95% CI] associated with rs2271036 (CC+CT *versus* TT genotypes) and rs2271037 (GT+GG *versus* TT genotypes) were 2.5 [1.2–3.8] (*p* = 0.007) and 2.3 [1.5–3.5] (*p* = 1.3×10^−4^) in the combined total sample, respectively.

A more thorough analysis showed that these associations were ethnic-specific as only significant in the Caucasian sub-group (C-Eur) with increased ORs (3.5 [1.8–6.6], *p* = 1.1×10^−4^ for rs2271036 and 3.1 [1.7–5.8], *p* = 2.1×10^−4^ for rs2271037 (Table 3 lines C-Eur).

Finally, Among PE cases, rs2271036 and rs2271037 showed no significant association with PE severity (*p* = 0.5 and *p* = 0.3, respectively), time of onset (*p* = 0.5 and *p* = 0.4, respectively), or with the proportion of neonates SGA (*p* = 0.2 and *p* = 0.2, respectively). Note that because rs2271036 and rs2271037 SNPs are almost in perfect linkage disequilibrium in the Caucasian sample (r^2^ = 0.93) no multivariate analyses, i.e. including the 2 SNPs in the same model, were conducted.

## Discussion

Here, we report for the first time a highly significant association between two SNPs located in the *CORIN* gene (rs2271036 and rs2271037) and PE in a large sample of 571 matched patients. This association is specifically observed in Caucasian patients originated from Europe.

To date, more than 150 genes have been described in relation to PE or haemolysis, elevated liver enzymes and low platelets (HELLP) syndrome, a thrombotic microangiopathy that can complicate PE [20]. The genes concern such various biological processes as immune, cell communication, metabolic processes or inflammation for example. Recently, two meta-analysis of more than 500 genetic associations identified only a few genetic variants that remained significantly associated with PE [21], [22]. These genetic variants are *F5* rs6020 and rs6025, *F2* rs1799963, *ACE* rs4646994, *AGT* rs699, *AGTR1* rs5186, *CTLA4* rs231775, *LPL* rs268 and *SERPINE1* rs1799889. The ORs [95% IC] range from 1.17 [0.99–1.40] (*ACE* rs4646994) or 1.17 [1.03–1.33] (*SERPINE1* rs1799889) to 2.42 [1.25–4.68] (*LPL* rs268) [21], [22].

The *CORIN* gene was first described by Pan *et al* concomitantly in human and mice [13]. The human corin gene spans 244 kb, and consists of 22 exons and 21 introns. The functional roles of corin domains have been studied with deletion mutant constructed recombinant proteins [15]. The authors showed that Frizzled 1domain and LDLR repeats 1 to 4 are critical for corin pro-ANP processing activity. Interestingly, rs2271037 and rs2271036 SNPs are located immediately before and after exon 9, coding for LDLR4 domain. They are both located in highly conserved suggestive of a functional relevance. In addition, rs2271037 closely maps to a splicing site.

Corin deficiency was first described in hypertensive heart disease [14], [23], with experimental evidence for a causal relationship, since corin deficient mice displayed no detectable levels of ANP and increased atrial pro-ANP expression while wild-type mice, had spontaneous hypertension and exhibited cardiac hypertrophy [11]. In humans, two SNPs in near-complete linkage disequilibrium (T555I/Q568P, exon 12) were found to be independently associated with increased risk for hypertension as well as left ventricular hypertrophy in the presence of untreated hypertension specifically in African-American [14], [23]. These results were found in a discovery sample and confirmed in a replication sample composed of White, Black and Hispanic participants. The amino-acid changes were non-conservative and located in the Frizzled 2 domain, in a highly conserved sequence across species, and displayed a reduced pro-ANP processing activity compared to that of wild-type as a covariant T555I/Q568P in human embryonic kidney (HEK) 293 cells and murine HL-1 cardiomyocytes [24]. Surprisingly, we did not find any association between SNPs in the *CORIN* gene and PE in patients from Sub-Saharan Africa, a high-risk population for PE, especially very early-onset PE [25]. In our study, we only sequenced exons and exons-flanking intronic regions, which does not exclude the existence of such SNPs elsewhere in *CORIN* introns. Interestingly, rs2271037 affects a very highly conservative site when comparing *CORIN* sequence between species; the nucleotide differs only in humans and the SNP is associated to a human-specific disease, suggesting subsequent functional impact of the SNP on corin expression or activity. This could be due to impaired splicing as suggested by the close position of the SNP near a splicing site.

The strong association of rs2271036 and rs2271037 SNPs with PE is suggestive of a contributive role of corin in PE. This statement is in agreement with 2 series of observations. First, corin deficient pregnant mice demonstrate late-gestation proteinuria and hypertension, mimicking human PE phenotype [11], [12]. Abundant corin mRNA expression was observed in the decidual cells of the mouse uterus, close to the implantation site of the embryo [10], [12]. Cui *et al* also reported corin mRNA and protein expression in uterus samples from pregnant women, which were lower in patients with PE than normal pregnancies [12]. Kaitu’u-Lino *et al* also showed that corin is expressed in first trimester human implantation sites and is up-regulated with endometrial decidualization [26]. Second, Cui *et al* described for the first time two *CORIN* mutations in 3 Han Chinese patients, after sequencing *CORIN* exons in 56 PE patients and 108 normal pregnant women [12]. K317E and S472G mutations are located in LDLR2 and Frizzled 2 domains respectively, which also have critical functional role in pro-ANP processing activity, and displayed markedly reduced corin pro-ANP processing activity in HEK293 cells, without affecting its expression [12], [15].

This is the first report of a highly significant association between two SNPs in the *CORIN* gene and PE in Caucasian women from Europe, and also the strongest association between 2 SNPs and PE described so far. These findings reinforce the probability of a critical role of corin in PE pathophysiology at the uteroplacental interface. Likewise, understanding how these SNPs can affect corin uterine expression and/or function are questions to be investigated. Further propective studies are also needed to confirm the strength of these associations and their potential to screen for high risk of preeclampsia.
